# Tracheal rupture after misplacement of Sengstaken-Blakemore tube

**DOI:** 10.11604/pamj.2016.23.55.8890s

**Published:** 2016-02-29

**Authors:** Adriá Rosat, Emilio Martín

**Affiliations:** 1 Department of General Surgery, Hospital Universitario Nuestra Señora de Candelaria, Ctra, Del Rosario 145, 38010 Sta, Cruz de Tenerife, Spain; 2 Department of Thoracic Surgery, Hospital Universitario Nuestra Señora de Candelaria, Ctra, Del Rosario 145, 38010 Sta, Cruz de Tenerife, Spain

**Keywords:** Tracheal rupture, hematemesis, Sengstaken-Blakemore

## Image in medicine

A 62-year-old man was admitted in our hospital's emergency room for hematemesis. He had a known history of alcoholic liver cirrhosis with severe esophagitis and previous episodes of hematemesis. The endoscopy showed no varix or perforation but an active bleeding from multiple ulcerations which cannot be controlled. An attempt to achieve hemostasis by balloon tamponade was undertaken using a Sengtaken-Blakemore tube. During insertion of the catheter, the patient became agitated and respiratory distress was noted when all ballons were inflated. The balloon were immediately deflated and a chest X-ray was performed, showing the tube in the right bronchus airway (A), so it was withdrawn. Right pneumothorax appeared and was treated with an intercostal drainage. The patient required orotracheal intubation and a CT scan was performed to show the rupture level (B). The patient underwent surgery and the trachea was successfully repaired. The Sengstaken-Blakemore was withdrawn and esophagoscopy was performed showing no bleeding. The patient was extubated 12 hour after the intervention. Endoscopic examination of the tracheal suture on the 11th postoperative day demonstrated normal healing and the patient was discharged two days later. The use of balloon tamponade for hemostasis in upper digestive tract has long been associated with numerous complications. The most common complication is rupture of the esophagus but respiratory complications can result from laryngeal or tracheal ischemia due to compression. Iatrogenic acute tracheal rupture is the less frequent of them. The presence of respiratory distress before the balloons were inflated could have avoided the misplacement.

**Figure 1:**
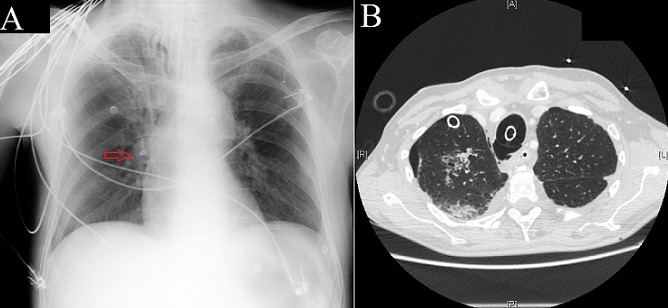
A): chest x-ray showing Sengstaken-Blakemore tube in the right bronchus just after balloon were deflated (arrow points the tip); B): chest CT scan showing right pneumothorax with chest tube, pneumomediastinum and orotracheal tube with the tracheal rupture level

